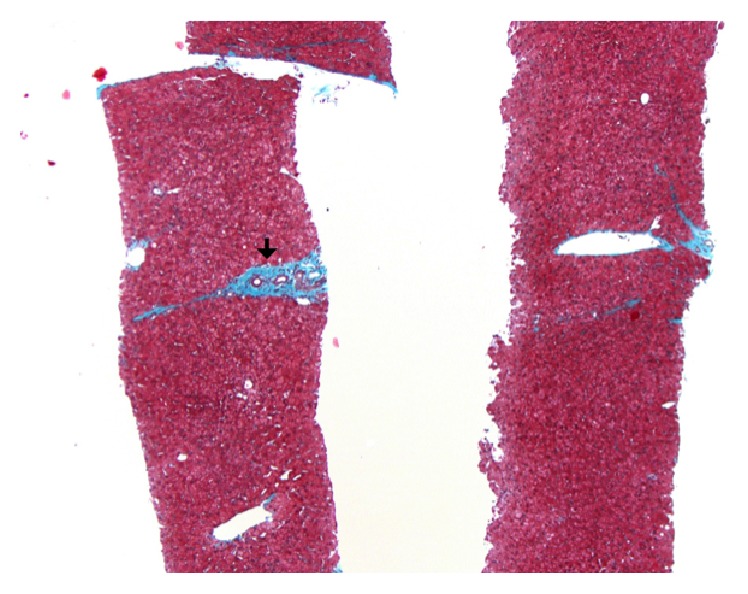# A Curious Case of Iron-Deficiency Anemia

**DOI:** 10.1155/2016/8954308

**Published:** 2016-04-03

**Authors:** Seth Shaffer, Mayur Brahmania, Hemant Shah

**Affiliations:** ^1^Department of Medicine, Queen's University, Kingston General Hospital, Kingston, ON, Canada K7L 2V7; ^2^Toronto Centre for Liver Diseases, Toronto General Hospital, University Health Network, Toronto, ON, Canada M5G 2C4

## Abstract

A 49-year-old Brazilian male presented to the emergency department with a five-day history of abdominal pain, dark stools, and syncope. Physical examination did not reveal any melena on digital rectal examination and there were no stigmata of chronic liver disease. Laboratory results showed hemoglobin of 47 g/L, MCV of 80 fL, and ferritin of 6 ng/mL. Liver enzymes and liver function tests were normal. Abdominal ultrasound showed a cirrhotic liver with splenomegaly and varices suggestive of portal hypertension. His past history was significant for cirrhosis based on a previous variceal bleed but a workup for chronic liver disease was negative and a liver biopsy did not show steatosis, fibrosis, or cirrhosis. A gastroscopy in this admission showed large esophageal varices without high-risk stigmata and no overt bleeding was seen. A colonoscopy was subsequently completed to the terminal ileum and was normal aside from a 5 mm sessile polyp in the descending colon.

## 1. Case Presentation

**Figure 1:**
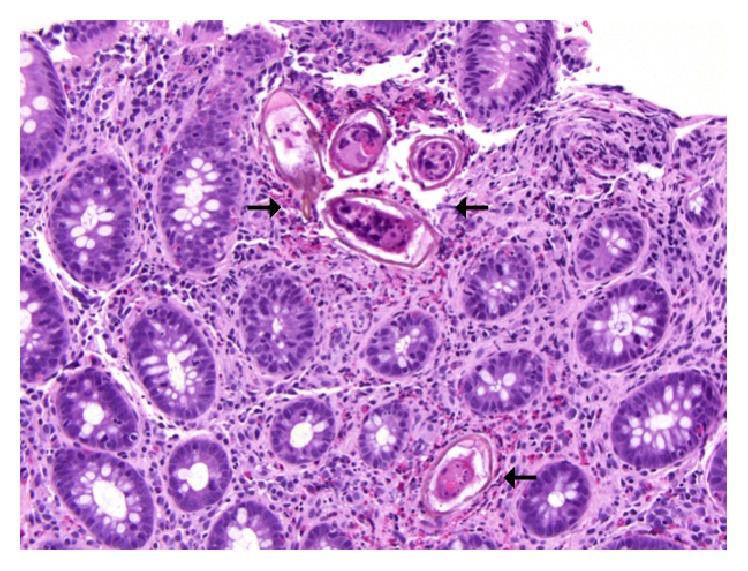


A 49-year-old Brazilian male presented to the emergency department with a five-day history of abdominal pain, dark stools, and syncope. His past medical history was significant for cirrhosis, based on a previous esophageal variceal bleed; but a workup for chronic liver disease was negative and a liver biopsy at that time did not show steatosis, fibrosis, or cirrhosis. Physical examination did not reveal any melena on digital rectal examination and there were no stigmata of chronic liver disease. Laboratory results showed hemoglobin of 47 g/L, MCV of 80 fL, and ferritin of 6 ng/mL. Liver enzymes and liver function tests were normal. Abdominal ultrasound showed a cirrhotic liver with splenomegaly and varices suggestive of portal hypertension. Gastroscopy showed large esophageal varices without high-risk stigmata and no overt bleeding was seen. A colonoscopy was subsequently completed to the terminal ileum and was normal aside from a 5 mm sessile polyp in the descending colon. Pathology of this polyp is located in Figure 1, under a hematoxylin-eosin stain, magnified 200 times. Arrows are pointing to numerous eggs in the stroma of the colonic mucosa, surrounded by eosinophil-rich inflammatory infiltrate. The eggs are large and have a characteristic shape, with a prominent lateral spine. A repeat liver biopsy was undertaken and is shown in Figure 2, under Masson trichrome stain, magnified 50 times. The arrow is pointing to portal fibrosis, with no significant architectural distortion or nodularity typical of cirrhosis.

## 2. Discussion

The patient was diagnosed with chronic schistosomiasis causing iron-deficiency anemia and noncirrhotic portal hypertension. Schistosomiasis is an enteropathogenic disease caused by parasitic trematode blood flukes from the genus* Schistosoma* and is transmitted by penetrating the intact epidermis and entering the bloodstream [1]. It is estimated to affect 240 million individuals worldwide and is a frequent cause of morbidity and mortality in Egypt, Sudan, Brazil, China, and the Philippines [2]. The eggs cause pathologic damage through inflammation and eventually form nodules and polyps. Schistosomiasis may also affect hepatic parenchyma as trapped eggs in the liver cause granulomatous inflammation resulting in fibrosis, which can occlude the portal veins and cause portal hypertension [2]. While the underlying hepatic function remains preserved, ascites and varices can develop [2]. Other gastrointestinal manifestations include chronic abdominal pain, diarrhea, and bloody stools. Lab values often reflect manifestations of schistosomiasis resulting in iron-deficiency, eosinophilia, and hypoalbuminemia with no biochemical evidence of cirrhosis. Ultrasound will show features of cirrhosis with a ring of concentric fibrosis surrounding the portal vein vasculature known as a “bull's eye lesion” [3]. Liver biopsy may show whitish plaques, termed “clay pipe-stem fibrosis” as the portal vein and tributaries become fibrotic and appear similar to pipe-stems. Diagnosis is by the detection of schistosomal eggs in the stool or urine [1]. Biopsies of the rectal mucosa or liver are also used as an alternative, as well as immunologic assays. The treatment of schistosomiasis is Praziquantel, which is active against all species in a single dose [1]. The patient was treated successfully with Praziquantel and had no further bleeding; however, his liver disease at this point was irreversible.

## Figures and Tables

**Figure 2:**